# Bachmann's bundle pacing: toward physiologic atrial activation

**DOI:** 10.3389/fcvm.2026.1895174

**Published:** 2026-07-29

**Authors:** Ke Zhang, Mary Lyn Bou Chaaya, Talia Hassoun, Nicholas Y. Tan, Fatima M. Ezzeddine

**Affiliations:** 1 Department of Cardiovascular Medicine, Mayo Clinic, Rochester, MN, United States; 2 Department of Biology, American University of Beirut, Beirut, Lebanon

**Keywords:** atrial fibrillation, Bachmann's bundle, cardiac pacing, cardiac resynchronization therapy, right atrium

## Abstract

Bachmann's bundle (BB) pacing is an emerging physiological atrial pacing strategy designed to preserve interatrial conduction and coordinated atrial activation. Conventional right atrial appendage pacing may produce non-physiological activation, contributing to atrial dyssynchrony, impaired left atrial function, and increased risk of atrial fibrillation. Because BB is the predominant pathway for right-to-left atrial conduction, targeted BB pacing can shorten the P-wave duration and improve atrial synchrony. Recent fluoroscopic, electrogram-guided, and P-wave morphology criteria have improved confirmation of BB capture. Available studies suggest that BB pacing is feasible and generally safe, with potential benefits in reducing the burden of atrial arrhythmias. However, the long-term clinical outcomes remain uncertain. Larger prospective studies are needed to standardize BB capture criteria, define optimal patient selection, and assess long-term outcomes.

## Introduction

Right atrial appendage (RAA) pacing remains the most widely used atrial pacing technique because of its technical ease and stable lead pacing and sensing parameters; however, it may promote nonphysiological atrial activation, interatrial dyssynchrony, and a high burden of atrial arrhythmias, prompting a growing interest in alternative physiological pacing sites beyond the RAA, including the interatrial septum, posterosuperior right atrium, and the Bachmann's bundle (BB) region (1, 2). Since conduction from the right atrium (RA) to the left atrium (LA) occurs predominantly via the BB, this structure has become an important target for atrial resynchronization (3). Recent interest in BB pacing is mostly due to its promise to achieve more physiological interatrial conduction, reduce interatrial dyssynchrony, and mitigate arrhythmogenic substrates linked to conventional pacing (1, 2). Although clinical evidence remains limited, emerging data suggest that BB pacing may offer advantages in selected patient populations, especially those with atrial conduction abnormalities or a high anticipated burden of atrial pacing (1).

## Anatomy of the Bachmann's bundle

**Figure 1:**
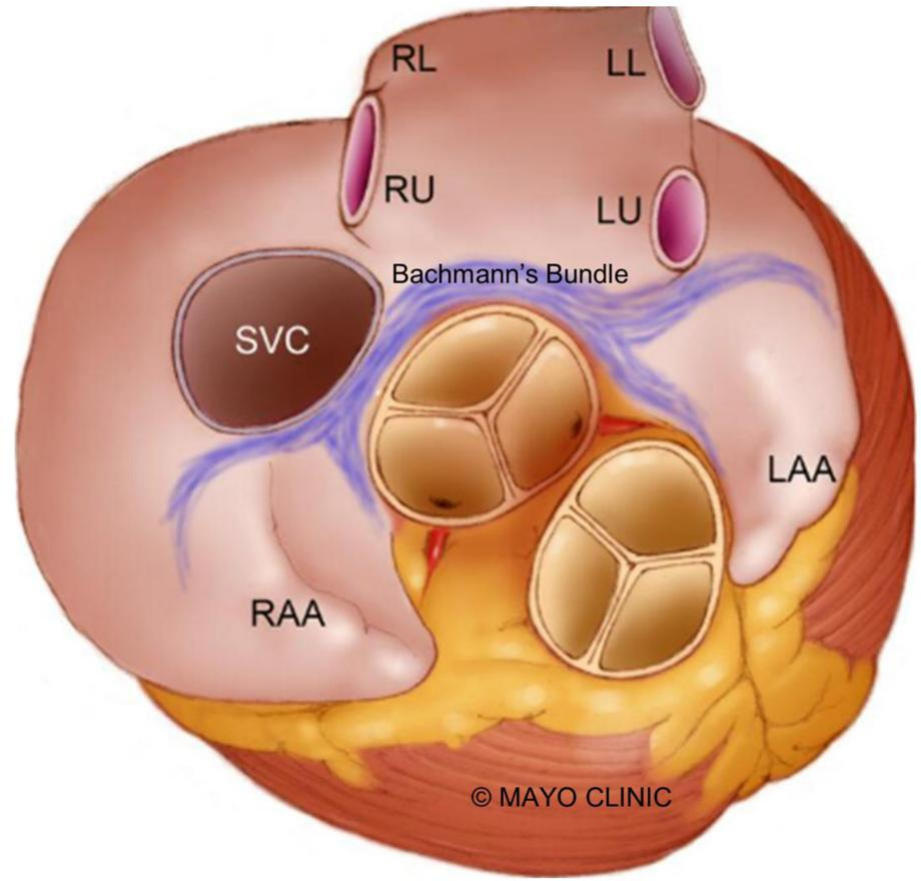
Bachmann's bundle anatomy. LAA, left atrial appendage, LL, left lower, LU, left upper, RL, right lower, RU, right upper, RAA, right atrial appendage, SVC, superior vena cava.

The BB is comprised of parallel-aligned strands of atrial myocardial fibers without a surrounding fibrous tissue sheath and can be grossly identified as a discrete bundle at the interatrial groove (septal raphe), which is separated by fatty tissue from the atrial wall (4). It extends bidirectionally to form leftward and rightward extensions, which bifurcate to connect the atrial appendages and blend into the musculature of the atrial walls (Figure 1) (4, 5). The rightward extension has a superior arm toward the cavoatrial junction near the crista terminalis and sinoatrial node, and an inferior arm to the RA vestibule. On the left, the bundle runs along the anterior LA wall around the left atrial appendage, with superior fibers along the left lateral ridge anterior to the left pulmonary veins and inferior fibers descending to the atrial vestibule (4, 5).

The BB is recognized as the principal preferential interatrial conduction pathway connecting the right and left atria. Earlier experimental studies suggested that BB fibers may possess electrophysiological characteristics intermediate between ordinary atrial myocardium and specialized conduction tissue, including relatively rapid conduction properties and distinct responses to autonomic stimulation and potassium arrest (6, 7). However, contemporary anatomical and electrophysiological studies indicate that the rapid conduction through the BB is primarily due to its highly organized myocardial architecture, characterized by parallel alignment of cardiomyocytes and anisotropic conduction properties, rather than the presence of a specialized conduction system analogous to the His-Purkinje network (8–11). These unique features support the role of the BB as a preferential interatrial conduction pathway during sinus rhythm (12). Impulses from the sinoatrial node spread through the RA and enter the adjacent BB, in which longitudinal fibers preferentially conduct signals to the LA. This produces early anterior LA breakthrough and promotes coordinated activation of both atria (13, 14).

## Patient selection

Atrial arrhythmias are common in patients with interatrial block (IAB). IAB is defined as P wave duration (PWD) greater than 120 ms on the surface electrocardiogram and may present with notched P waves in leads I, II, and III, terminal negativity in V1, and biphasic P waves in the inferior leads (15, 16). IAB is independently associated with incident atrial fibrillation (AF) and flutter (17). The underlying mechanism may involve increased temporal dispersion of atrial refractoriness, which broadens the inducible window during which ectopic impulses can initiate AF (18–20). Animal experiments showed that the mid-portion of BB with longer effective refractory period was the vulnerable region to develop re-entry and turn premature beats into AF (21). In humans, pacing at the BB was shown to decrease atrial conduction delay and prolong refractoriness at vulnerable sites, preventing the initiation of AF (22). Therefore, BB pacing has been proposed as a strategy to enhance interatrial conduction, shorten interatrial conduction time, and improve interatrial synchrony, potentially improving atrial hemodynamics and reducing the risk of AF (23). As such, the best candidates for this pacing approach are patients with atrial conduction delay who require frequent atrial pacing and are prone to atrial arrhythmias (1).

## Delivery sheaths and leads

Compared with the trabeculated inner face of the RAA, the smooth endocardial surface at the BB region requires active fixation to provide stable lead attachment. Traditionally, BB pacing relied primarily on fluoroscopic guidance for lead placement, therefore, any type of active fixation pacing lead was considered suitable (1). This included fixed-helix or extendable/retractable-helix stylet-driven leads, or sheath-delivered lumenless leads, delivered through a pre-shaped or deflectable delivery catheter (1, 24). With the increased adoption of electrogram-guided BB, a thin-caliber, non–stylet-driven, exposed-helix lead (Model 3830-59; Medtronic, Minneapolis, MN) delivered through a fixed-curve or single-plane deflectable sheath (Models C315-S4, C304-59; Medtronic, Minneapolis, MN) combination has been shown to be effective for electrogram mapping and BB pacing (25).

## Landmarks for lead placement

Initial localization of the BB region is performed under fluoroscopic guidance. With left axillary venous access, the sheath will be oriented towards the interatrial septum in left anterior oblique (LAO) projection after the delivery sheath is advanced into the RA (25). Gentle withdrawal of the sheath will bring the delivery system from the fossa ovalis back to the superior vena cava (SVC)-RA junction, which is characterized by leftward and superior movement of the tip in the LAO view (25). Radiopaque contrast injection can help with clarifying the relative location of SVC and RA roof. Subsequently, LAO caudal view is used to provide a bottom-up clock face view of the SVC, and BB electrograms are typically identified between the 5 and 2 o'clock positions (25, 26).

Subsequent target refinement relies on electrogram mapping and pace mapping (25, 27). Unipolar mapping is performed through an electrogram acquisition system. The BB electrogram shows a far-field signal occurring near the onset of the surface P wave, corresponding to RA activation, followed by high-frequency, low-amplitude BB potentials lasting approximately 20–80 ms (25, 26). Pacing at this site will generate P waves with normal axis and shorter duration compared with sinus rhythm.

## Criteria for optimal Bachmann's bundle pacing

Comprehensive P-wave morphology criteria for BB pacing includes: 1) vector similar to normal sinus rhythm with upright P-waves in leads I, II, III, and aVF; biphasic or negative P-wave in lead V1; 2) peaked and symmetric P-waves in the inferior limb leads with the paced P-wave amplitude typically greater than the sinus P-wave amplitude; 3) PWD narrowing compared with sinus PWD, typically by >10 ms if baseline IAB is present (27, 28).

Isoelectric capture is defined as an isoelectric interval between the pacing stimulus and the onset of a narrow, normal-appearing surface P wave (26). While the increased stimulus-to-P-wave latency with decrementing pacing output makes it resemble “selective” capture as in HIS/left bundle branch pacing (LBBP), the presence of isoelectric capture is instead due to lead placement higher in the SVC, where the impulse travels down the SVC sleeve before activating the RA, creating the isoelectric interval (26). Recent data demonstrated that pacing at the center of the BB did not produce an isoelectric interval in any case, whereas pacing superior to the BB region produced an isoelectric interval in 85% of cases (29). Isoelectric capture possesses risk of programming challenges as the isoelectric interval must be accounted for in the AV interval, micro-dislodgment, and perforation; therefore, lead repositioning should be considered when it is observed (26).

**Figure 2:**
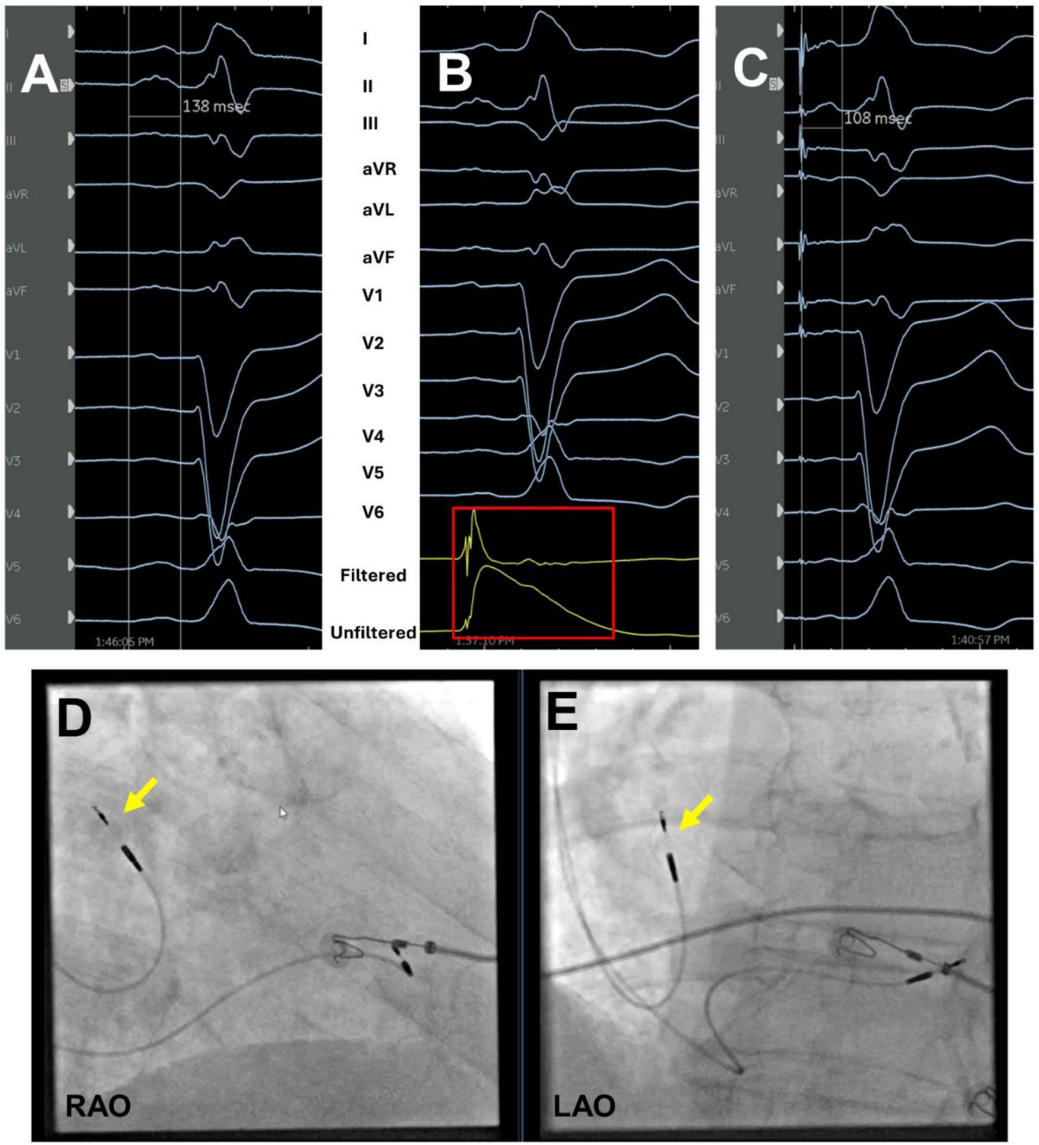
Example of Bachmann's bundle pacing. **(A)** Baseline interatrial block with a P wave duration of 138 ms; **(B)** current of injury post-screw; **(C)** P-wave morphology after BB pacing with a duration of 108 ms; (**D** and **E**) right anterior oblique (RAO) and left anterior oblique (LAO) fluoroscopic views showing BB pacing lead position (yellow arrow).

After active fixation, a relaxed open J-shaped lead configuration, with the ring electrode positioned posterior to the tip electrode, has been shown to be critical for adequate sensing (Figure 2) (25). To achieve this, residual torque can be released before sheath slitting with a deflectable system by twisting the lead body approximately half a turn counterclockwise to open the J shape, whereas when the pre-shaped C315 sheath is being used, the sheath must first be slit in order to release the lead torque and confirm adequate lead slack (25).

An important limitation of contemporary BB pacing is the absence of universally accepted electrophysiological criteria analogous to those established for His bundle pacing or left bundle branch pacing. Consequently, confirmation of BB capture in most studies relies on a combination of lead location, fluoroscopic landmarks, local electrogram characteristics, paced P-wave morphology, and shortening of P-wave duration. While these findings support engagement of the BB, they do not definitively prove selective capture of the BB. Recent investigations have emphasized the distinction between the anatomical BB, electrogram-defined BB targets, and fluoroscopically guided BB-area pacing sites, highlighting that these entities should not be considered interchangeable. Subramanian et al. demonstrated the feasibility of electrogram-guided BB-area pacing using recorded BB potentials and transitions between nonselective and selective capture, while acknowledging the ongoing challenge of establishing definitive capture criteria (30). Likewise, emerging mapping and anatomical studies suggest that many previously described “BB pacing” implants may more accurately represent BB-area pacing rather than proven capture of the discrete anatomical bundle.

## Success rate

Before the P wave morphology criteria for BB pacing were proposed, early implants were primally guided by fluoroscopy. In a retrospective study evaluating atrial arrhythmia outcomes of BB pacing, only 55% of the non-specific high RA septal pacing met all three P wave morphology criteria (27). Overall BB pacing is feasible, with success rates ranging from 72.4% to 91.3% (30–33). Procedural and fluoroscopy times were acceptable, with reported procedural times of approximately 67.5–71.8 min and fluoroscopy times of 9.6–12.5 min (30, 33). In a direct comparison of physiologic BB/LBBP vs. conventional RAA/right ventricular apical pacing, overall procedure and fluoroscopy times were similar, while the physiologic pacing strategy required longer procedure and fluoroscopy times after excluding patients who had >2 leads implanted (31). Lead parameters for BB pacing were generally clinically acceptable, though some studies showed lower P-wave sensing, higher pacing thresholds, and higher impedance in BB pacing in contrast to RAA pacing (27, 31). The longest reported follow-up duration comes from the study by Infeld et al., in which atrial leads used in BB pacing demonstrated stable parameters over 58 months after implantation, although pacing thresholds and impedance were higher relative to RAA pacing (27).

## Complications

The currently available BB pacing studies suggest an overall acceptable safety profile, with complications primarily related to acute lead stability, capture durability, and technical implantation challenges. In the retrospective cohort by Infeld et al., atrial lead revision was uncommon, occurring in 2 of 134 patients who underwent BB pacing, with rate comparable to those observed with RA septal and RAA pacing (27). Subramanian et al. reported acute dislodgement in 2 of 32 successful BB pacing implants, with one patient developing a later threshold increase; no pericardial effusion, infection, or late dislodgement was observed (30). Similarly, in the hypertrophic cardiomyopathy cohort, one acute lead dislodgement and one acute threshold rise occurred, without late dislodgement or device infection during follow-up (33). These events likely reflect the technical difficulty of targeting a small, anatomically variable superior septal/atrial roof region (26), where lead fixation, adequate slack, and consistent BB engagement may be challenging. Long-term lead durability and complication rates remain incompletely defined.

## Outcomes of Bachmann's bundle pacing

Table 1 provides a summary of the procedural and clinical outcomes reported for high right atrial septal pacing and BB pacing over time. BB pacing may have beneficial effects on both hemodynamics and the occurrence of AF. In 2001, Bailin et al. reported in a multicenter randomized controlled trial comparing BB pacing with RAA pacing in patients with paroxysmal AF that patients with BB pacing had a significantly higher survival rate without chronic AF compared with patients with RAA pacing after 1 year of follow-up (34). Later, Infeld et al. demonstrated that patients with BB pacing had lower atrial arrhythmia burden, recurrence, and *de novo* incidence compared to high RA septal and RAA pacing groups (27). Recently, Wu et al. conducted a prospective, randomized, single-center study on 72 patients with sinus node dysfunction and found greater improvement in atrial synchrony, more favorable trends in ventricular dimensions at 3-month follow-up, and a lower atrial arrhythmia burden at 12-month follow-up in the BB pacing group compared with the RAA pacing group, while no difference in the rate of new-onset AF was identified (32). Similar low AF/atrial tachyarrhythmia (AT) burden was found by Subramanian et al., who studied accelerated BB pacing in patients with non-obstructive hypertrophic cardiomyopathy and found that BB pacing was associated with lower NT-proBNP levels and improvement in diastolic function and exercise capacity, compared to RAA pacing (33). The inconsistent reduction in *de novo* AF observed across studies might be related to heterogeneity in study populations, atrial pacing burden, comorbidities, and follow-up duration. However, whether these rhythm benefits translate into more clinically meaningful outcomes—such as fewer cardioversions or AF-related hospitalizations, lower stroke risk, reduced heart failure events, or improved survival—remains uncertain and requires larger prospective studies with longer follow-up.

**Table 1:** Summary of procedural and clinical outcomes of Bachmann's bundle pacing and high right atrial septal pacing.

Studies	Design	Patient population	Study arms	Lead position defined by	P-wave morphology criteria	Follow-up duration (months)	Major clinical outcomes
Bailin et al. (34)	Multicenter, RCT	Paroxysmal AF (100%), SND (68%)	BBp (*n* = 63) vs. RAAp (*n* = 57)	Fluoroscopy	NA	12 ± 8	BBp reduced PWD and progression to permanent AF.
Roithinger et al. (38)	Prospective, single center, within-patient observational study	Post-SVT ablation (100%), no structural heart disease (100%)	Pacing from BB (*n* = 28) vs. CS ostium (*n* = 28) vs. high lateral RA (*n* = 28) vs. RAA (*n* = 8)	Fluoroscopy	NA	NA	Total atrial activation time was shortest during pacing from the presumed BB insertion site: 74 ± 18 ms, compared with CS ostium 94 ± 18 ms and high RA 118 ± 18 ms.
Goette et al. (39)	Single center, RCT	Post-CABG patients without prior AF/flutter (100%)	No pacing (*n* = 50) vs. lateral RAp (*n* = 60) vs. BBp (*n* = 51)	Fluoroscopy	NA	NA	Overall incidence of postoperative AF was not significantly different among groups. Compared with lateral RAp, BBp reduced AF incidence, produced significantly shorter PWD, and had lower pacing thresholds.
Hermida et al. (40)	Single center, RCT	Paroxysmal AF (59%), SND (50%)	RASp (mix of high, mid, and low RAS) (*n* = 28) vs. RAAp (*n* = 28)	Fluoroscopy	NA	5 ± 3	RASp reduced PWD, interatrial and AV conduction time, left atrial electromechanical delay, and AF episodes.
Padeletti et al. (41)	Multicenter, RCT	Paroxysmal AF (100%), SND (70%)	RASp (mix of high, mid, and low RAS) (*n* = 148) vs. RAAp (*n* = 150)	Fluoroscopy	NA	6	Neither RASp alone nor preventive pacing algorithms alone significantly reduced AT/AF burden compared with controls. AF prevention algorithm plus RASp reduced AT/AF burden.
Nigro et al. (42)	Single center, RCT	Myotonic dystrophy type 1 (100%)	BBp (*n* = 14) vs. RAAp (*n* = 16)	Fluoroscopy	NA	12	BBp was safe and feasible but did not significantly reduce number of AF episodes or AF duration compared with RAAp. Lead parameters remained stable during follow-up.
Infeld et al. (27)	Retrospective, single center, observational study	IAB (100%), atrial pacing >20% (100%), paroxysmal AF (44%)	BBp (*n* = 134) vs. non-specific RASp (*n* = 107) vs. RAAp (*n* = 108)	Fluoroscopy + Paced P-wave morphology	Similar P-wave vector to SR; Peaked and symmetric P-waves in the inferior leads; PWD narrowing compared to SR, >10 ms in IAB.	62 ± 38	BBp was associated with lower AF/AT burden, recurrence, and *de novo* incidence than RASp or RAAp.
Vedage et al. (43)	Retrospective, single center, observational study	Non-ischemic CM (60%), persistent AF (19%), paroxysmal AF (10%)	Baseline SR vs. temporary RAAp vs. final BBp (*n* = 21)	Fluoroscopy + Paced P-wave morphology	Narrowing of the PWD; Inferior P-wave axis; Increased P-wave amplitude in lead II compared with SR.	NA	BBp shortened PWD, increased lead II voltage, shortened PR interval, lengthened PR segment, and preserved normal P-wave axis vs. RAAp.
Watanabe et al. (31)	Retrospective, single center, observational study	AF (28%)	BBp + LBBAP (PP) (*n* = 32) vs. RAAp + RVAP (CP) (*n* = 64)	Fluoroscopy + Paced P-wave morphology	Paced P-wave axis resembled SR; Inferior-lead P-waves were peaked and symmetric; Paced PWD was narrower than SR, >10 ms with baseline IAB.	2 ± 2	HRASp was feasible and safe vs. RAAp, achieved BBp in 72%, and combined well with LBBAP for practical physiologic dual-chamber pacing.
Subramanian et al. (30)	Prospective, single center, observational study	IAB (100%), paroxysmal AF (19%)	BBp (*n* = 36)	Fluoroscopy + BB potential + Paced P-wave mprphology	Paced PWD shorter (>20 ms) than the SR; Normal P-wave axis; Peaked, symmetric, upright P-waves without notching in the inferior leads.	20 ± 6	Electrogram-guided BBp was feasible and safe, successful in 88.9% of patients, and had stable lead performance during follow-up.
Subramanian et al. (33)	Single center, randomized crossover trial	Non-obstructive HCM/HFpEF (100%) IAB (100%)	Successful BBp (*n* = 42) randomized to DDDR vs. no atrial pacing VVI, then crossover Historicl cohort of RAA_p_ (*n* = 26)	Fluoroscopy + BB potential + Paced P-wave morphology	Paced PWD ≥20 ms shorter than SR and positive; Symmetric P-waves (without notching) in the inferior leads.	19 ± 3	BBp improved NT-proBNP, functional capacity, diastolic function, AA burden, and diuretic requirement vs. pacing off or RAAp in nHCM-HFpEF with IAB.
Wu et al. (32)	Single center, RCT	SND (100%)	BBp (*n* = 37) vs. RAAp (*n* = 35)	Fluoroscopy + Paced P-wave morphology	Paced PWD 20 ms shorter than native PWD; Isoelectric or negative P-wave in lead I and aVL, positive in leads II, III, and aVF, and a biphasic pattern in lead V1	12	BBp shortened PWD and interatrial mechanical delay, and lowered AA burden compared to RAAP in patients with SND.
Yoshimoto et al. (44)	Single center, observational study	Symptomatic bradycardia (100%)	BBp (*n* = 10) vs. RAAp (*n* = 10) vs. SR (*n* = 13)	Fluoroscopy + Paced P-wave morphology	Criteria by Infeld et al. (27)	NA	BBp preserved biatrial mechanical synchrony compared to SR, with shorter PWD and less IACD than RAAp.
Alzahrani et al. (45)	Retrospective, single center, observational study	Pacing indication with prior infection, high infection risk, limited life expectancy and low anticipated pacing burden (100%)	Leadless BBp attempts (*n* = 17)	Fluoroscopy + Paced P-wave morphology	Upright inferior P-waves and biphasic/negative V1 Peaked, symmetric inferior P-waves with paced amplitude >SR; PWD shortened by ≥10 ms vs. SR.	6 ± 4	Leadless atrial pacing near the BB was associated with acceptable procedural performance.
Tan et al. (46)	Prospective, single center, within-patient observational study	Paroxysmal AF (65%), persistent AF (23%), IACD (58%)	SR vs. BBp vs. RAAp Successful BBp (*n* = 24)	Fluoroscopy + Paced P-wave morphology	Similar p-wave axis as in SR with positive P-waves in I, II, III and aVF, and biphasic/negative P-wave in V1; Greater paced p-wave amplitudes than in SR in the inferior leads; Shorter paced PWD than compared to SR.	NA	BBp shortened PWD compared to SR and RAAp, but did not reduce acute LAP overall. In patients with IACD, BBp showed lower LAP than RAAp.
Yamashita et al. (29)	Prospective, single center, cross-sectional study	Paroxysmal AF (100%)	Intra-procedural pace mapping at 5 RA sites during AF ablation (*n* = 20)	Fluoroscopy + Paced P-wave morphology	P-wave axis equivalent to SR; P-wave voltage in inferior leads higher than SR; PWD >10 ms shorter than SR; No evident isoelectric interval during capture threshold testing.	NA	EAM/ICE-confirmed BBp showed SR-like P-wave axis, higher inferior-lead voltage, shorter PWD, and no isoelectric interval, defining reliable BB capture criteria.

AA, atrial arrhythmia; AF, atrial fibrillation; AT, atrial tachycardia; AV, atrioventricular; BB, Bachmann's bundle; BBp, Bachmann's bundle pacing; CM, cardiomyopathy; CP, conventional pacing; CS, coronary sinus; EAM, electroanatomical mapping; HCM, hypertrophic cardiomyopathy; HFpEF, heart failure with preserved ejection fraction; HRASp, high right atrial septal pacing; IAB, interatrial block; IACD, interatrial conduction delay; ICE, intracardiac echocardiography; LA, left atrium/left atrial; LAAp, left atrial appendage pacing; LAP, left atrial pressure; LBBAP, left bundle branch area pacing; LRAp, low right atrial pacing; ms, milliseconds; NA, not available; nHCM, non-obstructive hypertrophic cardiomyopathy; NT-proBNP, N-terminal pro-B-type natriuretic peptide; PES, programmed electrical stimulation; PM, pacemaker; PP, physiological pacing; PWD, P-wave duration; RA, right atrium/right atrial; RAp, right atrial pacing; RAAp, right atrial appendage pacing; RASp, right atrial septal pacing; RCT, randomized controlled trial; RVAP, right ventricular apical pacing; SND, sinus node dysfunction; SR, sinus rhythm; SVT, supraventricular tachycardia; VP, ventricular pacing.

## Other physiological atrial pacing techniques

The adoption of BB pacing has been challenged by difficulties in consistent lead placement and uncertainty regarding BB capture criteria. Within the broader framework of physiological atrial pacing, alternative approaches have therefore gained increasing interest. Interatrial septal pacing and posterosuperior bundle pacing target regions that may provide more reproducible anatomical landmarks (35–37). These approaches seek to achieve similar goals of synchronized biatrial activation while potentially overcoming some of the technical limitations associated with BB pacing. Although early studies suggest favorable effects on atrial activation patterns and arrhythmia burden, comparative data remain limited, and the long-term clinical outcomes associated with these emerging physiological atrial pacing strategies have yet to be clearly defined.

## Conclusion

BB pacing offers an alternative to conventional atrial lead placement which promotes more physiologic atrial conduction and coordinated atrial activation. Its potential advantages may help mitigate AF progression. However, larger studies are needed to better define its long-term clinical benefits and establish procedural standardization.
